# Serum Calcium, Magnesium, Zinc and Copper Levels in Sudanese Women with Preeclampsia

**DOI:** 10.1371/journal.pone.0167495

**Published:** 2016-12-02

**Authors:** Abdelmageed Elmugabil, Hamdan Z. Hamdan, Anas E. Elsheikh, Duria A. Rayis, Ishag Adam, Gasim I. Gasim

**Affiliations:** 1 Faculty of Medicine, El Imam El Mahdi University, Kosti, Sudan; 2 Faculty of Medicine, Alneelain University, Khartoum, Sudan; 3 Faculty of Medicine, Omdurman University, Omdurman, Sudan; 4 Faculty of Medicine, University of Khartoum, Khartoum, Sudan; Yale School of Public Health, UNITED STATES

## Abstract

**Background:**

Although the exact pathophysiology of preeclampsia is not fully understood, several elemental micronutrient abnormalities have been suggested to play a contributory role in preeclampsia.

**Aims:**

To investigate the levels of calcium, magnesium, zinc and copper in women with preeclampsia.

**Subjects and Methods:**

A case—control study was conducted in Omdurman Maternity Hospital, Sudan, during the period of September through December 2014. The cases were women with preeclampsia while healthy pregnant women were the controls. The medical and obstetrics history was gathered using questionnaires. The serum levels of calcium, magnesium, zinc and copper were measured using atomic absorption spectrophotometer.

**Results:**

There was no significant difference between the two groups in their age, gestational age, parity and body mass index. Zinc and copper levels were not significantly different between the two groups. In comparison with the controls, women with preeclampsia had a significantly lower median (inter-quartile) serum calcium [7.6 (4.0─9.6) vs. 8.1 (10.6─14.2), mg/dl, P = 0.032] and higher levels of magnesium [1.9 (1.4─2.5) vs. 1.4 (1.0─1.9) mg/dl; P = 0.003]. In binary logistic regression, lower calcium (OR = 0.73, 95% CI = 0.56 ─ 0.95, P = 0.021) and higher magnesium (OR = 5.724, 95% CI = 1.23 ─ 26.50, P = 0.026) levels were associated with preeclampsia. There were no significant correlations between levels of hemoglobin and these trace elements.

**Conclusion:**

The current study showed significant associations between preeclampsia and serum levels of calcium and magnesium.

## Introduction

Preeclampsia is a common medical disorder affecting about 2–7% of pregnant women worldwide [1, 2] and can lead to unfavorable pregnancy outcomes such as increased maternal as well as perinatal morbidity and morbidity [3]. The etiology of preeclampsia remains ambiguous, albeit, reports that implicated placental defects and oxidative stress early during pregnancy in affected pregnancies [4, 5].

Micronutrients and trace elements play a pivotal role in metabolism and in the preservation of tissue function. Trace elements are important constituents of a number of antioxidants. Therefore they are integral part of a robust antioxidant that protect the cell from damage [6, 7].

Several elemental micronutrients abnormalities (calcium, magnesium, zinc, and copper) have been suggested to play a contributory role in preeclampsia [8–13]. However, the results of the reports describing the associations between serum concentration of zinc, copper, magnesium, calcium and preeclampsia varied greatly [2, 8, 14–17].

Therefore, research on trace elements and preeclampsia is of paramount importance for academicians, health planners as well as treating physicians. The findings of research on trace elements and preeclampsia is an evidence-base and could be implemented for prevention of preeclampsia e.g. calcium and zinc supplement [18]. In Sudan, preeclampsia/eclampsia is a major cause of maternal and perinatal morbidity and mortality [1, 19, 20]. We have previously shown that preeclampsia is associated with oxidative stress [21]. The current study was carried to investigate the levels of serum calcium, magnesium, zinc and copper in preeclampsia, to find their potential role in the etiology/pathogenesis of preeclampsia and to add to the previous research on preeclampsia in Sudan [21–23].

## Materials and Methods

A case ─ control study was conducted in Omdurman Maternity Hospital, Sudan during the period of September through December 2014. Omdurman Maternity Hospital is the largest tertiary maternity hospital in the region. It serves an area of 6 million people and there are 24913 deliveries per year (around 70 deliveries per day) [24].

The cases were women presented with preeclampsia (before receiving any medications), which was defined as hypertension (occurrence of systolic blood pressure ≥ 140 mm Hg or diastolic blood pressure ≥ 90 mm Hg after at least 20 weeks of pregnancy in a previously normotensive patient) plus at least 300 mg of protein in a 24-hour urine sample or a dipstick test result of at least 2+ [25]. Preeclampsia was further divided into mild and severe forms, according to a diastolic blood pressure of ≤110 mm Hg or > 110 mm Hg, respectively. Healthy pregnant women attended the prenatal care clinic of the same hospital during the study period were taken as the controls.

Women with diabetes mellitus, other endocrine disorder, and kidney disease were excluded from both cases and controls. After signing an informed consent a detailed history was obtained from all participants followed by physical and obstetrics examination. Weight and height were measured and body mass index was calculated and expressed as Kg/m^2^. An antecubital route was used to collect the blood samples from the antecubital vein of each of the patients and the controls. Sera were then centrifuged, separated and kept at 0–20°C till analysis. The serum calcium, magnesium, copper and zinc levels were measured using an atomic absorption spectrophotometer (SOLAAR, Atomic Absorption Spectrophotometer, Thermo Electron, Cambridge, UK) [26]. The quality assurance and assay accuracy were assured by using standard solutions for every ten test sample.

### Statistics

Data was analyzed using SPSS for Windows (version 20.0). A total sample size of 50 participants in each group was calculated to get the difference in the mean of the proposed variables (levels of trace elements) that would provide 80% power to detect a 5% difference at α = 0.05, with an assumption that complete data might not be available for 10% of participants. T-test and *X*^2^ tests were used to compare the normally distributed data (Mann-Whitney U if the data were not normally distributed) continuous variables and proportions between the two groups. Binary regressions were performed, where preeclampsia was the dependent variable and socio-demographic parameters (age, parity, education, job, residence), trace elements levels were the independent variables. Odds ratio (OR) and 95% confidence interval (CI) were calculated. Pearson/ spearman correlation was performed. P < 0.05 was considered statistically significant.

Research Board at the Department of Obstetrics and Gynecology, Faculty of Medicine, University of Khartoum, Sudan specifically approved this study.

All procedures performed in this study were in accordance with the ethical standards of the 1964 Helsinki declaration and its later amendments or comparable ethical standards. Informed consent was obtained from all individual participants included in the study.

## Results

While there was no significant difference between the two groups (50 women in each arm) of the study in their age, parity, gestational age and BMI, hemoglobin level was significantly lower in women with preeclampsia, Table 1.

**Table 1:** Comparing the mean (SD) of the basic characteristics between women with preeclampsia and controls

Variables	Preeclamptic women	Healthy controls	P
	(n = 50)	(n = 50)	
Age, year	28.6 (6.4)	28.6 (6.6)	0.988
Parity	2.3 (2.3)	2.7(2.8)	0.390
Gestational age, weeks	37.1(1.0)	36.8(1.0)	0.190
Body mass index, Kg/m^2^	29.0(5.0)	27.0(5.1)	0.139
Hemoglobin, g/dl	10.8(1.0)	11.5 (1.3)	0.018

**Fig 1:**
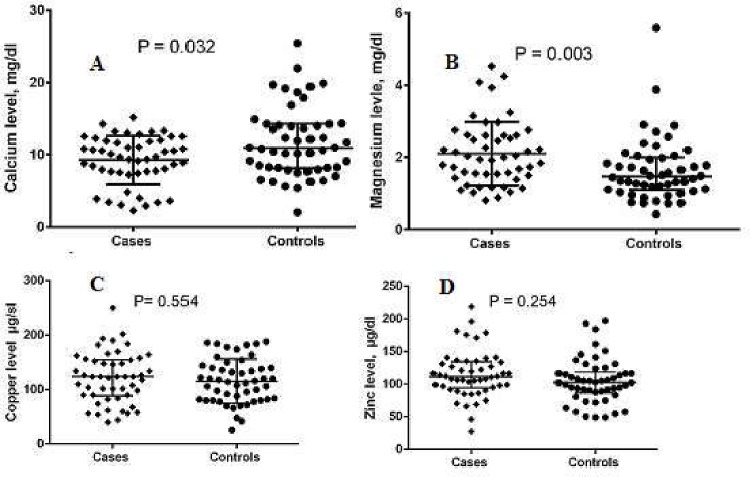
(A-D) Median (inter-quartile) of calcium, magnesium, zinc and copper levels women with in preeclampsia and controls.

In comparison with the controls, women with preeclampsia (four and 46 were mild and severe preeclampsia, respectively) had significantly lower calcium [7.6 (4.0─9.6) vs. 8.1 (10.6─14.2), mg/dl, P = 0.032] and higher levels of magnesium [1.9 (1.4─2.5) vs. 1.4 (1.0─1.9) mg/dl; P = 0.003]. There were no significant differences in the zinc, copper levels and zinc/ copper ratio between women with preeclampsia and the controls, Table 2, Fig 1A–1D.

**Table 2:** Comparing the median (inter-quartile of the trace elements between women with preeclampsia and controls

Variables	Preeclamptic women	Healthy controls	P
	(n = 50)	(n = 50)	
Calcium, mg/dl	7.6 (4.0─9.6)	8.1 (10.6─14.2)	0.032
Magnesium, mg/dl	1.9 (1.4─2.5)	1.4 (1.0─1.9)	0.003
Zinc, μg/dl	108.0 (91.6─131.7)	102.0 (82.8─124.0)	0.254
Copper μg/dl	111.6 (94.3─135.3)	103.6 (86.6─126.7)	0.554

In binary logistic regression, a low calcium (OR = 0.73, 95% CI = 0.56 ─ 0.95, P = 0.021) and a high magnesium (OR = 5.724, 95% CI = 1.23 ─ 26.50, P = 0.026) levels were associated with preeclampsia, Table 3.

**Table 3:** Binary logistic regression for the factors associated with preeclampsia

Variables	OR	95% CI	P
Age, year	0.98	0.79 ─1.21	0.854
Parity	1.06	0.69 ─ 1.64	0.768
Gestational age, weeks	1.61	0.67 ─ 3.85	0.283
Body mass index, Kg/m^2^	1.14	0.95 ─ 1.36	0.141
Hemoglobin, g/dl	0.93	0.46 ─ 1.85	0.841
Calcium, mg/dl	0.73	0.56 ─ 0.95	0.021
Magnesium, mg/dl	5.72	1.23 ─ 26.50	0.026
Zinc, μg/dl	1.00	0.99 ─1.00	0.154
Copper μg/dl	0.98	0.96 ─ 1.05	0.056

There were no significant correlations between gestational age, BMI, hemoglobin and calcium, magnesium, zinc and copper. There was no significant correlation between the investigated trace elements, Table 4.

**Table 4:** Correlations between gestational age, body mass index, hemoglobin and trace elements

Variable	S. calcium		S. magnesium		S. zinc		S. copper	
	*r*	*P*	*r*	*P*	*r*	*P*	*r*	*P*
Gestational age, year	0.023	0.820	0.	119 0.237	0.007	0.941	─ 0.094	0.351
Body mass index, Kg/m^2^	─ 0.187	0.230	─ 0.105	0.423	─ 0.051	0.698	─ 0.026	0.846
Hemoglobin, g/dl	0.031	0.794	0.272	0.018	0.014	0.224	0.102	0.381
Calcium, mg/dl	─	─	─ 0.077	0.446	─ 0.125	0.217	0.166	0.098
Magnesium, mg/dl	─ 0.077	0.446	─	─	0.086	0.395	─ 0.088	0.385
Zinc, µg/dl	─ 0.125	0.217	0.086	0.395	─	─	─ 0.135	0.182

## Discussion

The main findings of the current study were; significantly lower calcium, higher magnesium levels in women with preeclampsia, calcium and magnesium were associated with preeclampsia. Our finding (higher magnesium level) is in concurrence with recent reports, where Katz et al found higher levels of magnesium among 43 preeclamptic women who received magnesium sulphate (the patients in the current study were enrolled before commencement of any medications) [27]. Recently, Farzin and Sajadi reported significantly lower levels of calcium, magnesium and zinc in preeclamptic women compared with the controls [16]. Earlier, Jain et al observed significantly lower levels of calcium, magnesium and zinc in women with preeclampsia (25 with mild and 25 with severe preeclampsia) than the normal pregnant controls [8]. On the other hand, Negi et al observed decreased levels of magnesium zinc, copper in the umbilical cord blood of preeclamptic and eclamptic pregnancies. Yet, Vafaei et al observed no significant difference in the serum levels of calcium, magnesium and zinc levels in the 40 normotensive pregnancies (controls), 20 mild and 20 severe preeclamptic Iranian women [15]. Furthermore, an association between preeclampsia and hypocalciuria [28], decreased urine calcium to creatinine ratio [29], and low dietary milk intake [30] has also been established.

In the current study, although there was a low calcium and a higher magnesium level in preeclamptic women, calcium and magnesium levels showed no significant correlation. Interestingly, previous reports have delineated a competition between magnesium and calcium with one another for common binding sites on plasma protein molecules [31]. The interaction at molecule level and carrier protein level should be considered when the level of calcium, magnesium and albumin is investigated [32, 33]. Moreover, studies have shown that magnesium counteracts calcium-dependent release of acetylcholine at motor endplates [31]. Thus, magnesium may be regarded as a natural ‘calcium antagonist’

Magnesium is a trace element of paramount importance; it acts as a cofactor for a number of enzyme systems [34]. Magnesium is important for tone, contractility, and reactivity of blood vessels and, therefore, plays a pivotal role in the physiological regulation of blood pressure. This enhances the understanding of the therapeutic importance of magnesium in the treatment of preeclampsia [35].

Calcium has a major role in the rise of blood pressure; however, a balance between calcium and magnesium is needed to control blood pressure. This is because blood vessels need calcium to contract and magnesium to relax and open up. Therefore, magnesium can be considered as a calcium channel blocker by antagonizing the increase in the intracellular calcium concentration leading to vasodilatation [36–38].

It is worth to be mentioned, that previous studies have reported a link between low dietary calcium intake with increased incidence of preeclampsia. Likewise, supplementation of calcium has been reported to prevent preeclampsia [39, 40].

The current study showed no significant difference in copper level between cases and controls. Previous studies, reported an association between high maternal serum copper and preeclampsia [16, 27]. However, others linked the association with caeruloplasmin activity on a background of a raised serum copper rather than just a raised serum copper, probably secondary to impaired antioxidant enzymes [41].

In the current study, there were no significant correlations between hemoglobin and the trace elements we studied. This goes with our previous observation where we observed no correlations between zinc, copper and hemoglobin levels [26, 42].

Limitations of the present study includes that, the possible interactions between trace elements and their carrying vehicle were ignored. Measurement of serum albumin, serum caeruloplasmin and pH in future studies will help in better interpretations of the present findings.

## Conclusion

The current study findings indicate that the three trace elements; calcium and magnesium significantly associated with preeclampsia. On the other hand, zinc and copper were not associated with preeclampsia. Further researches that consider the complex relation between the trace elements and their carrier vehicle proteins are needed.

## Supporting Information

S1 Raw data (DOCX)
