# Supplementary material for: Serum Calcium, Magnesium, Zinc and Copper Levels in Sudanese Women with Preeclampsia
Source: PLoS One. 2016 Dec 2;11(12):e0167495. doi: 10.1371/journal.pone.0167495 (PMC5135106; doi:10.1371/journal.pone.0167495)
Supplement: S1 Raw data — (DOCX) [file pone.0167495.s001.docx]

| SN | New cod | calcium | magnesium | copper | zin | code | age | parity | GA | Case/control | controls | BMI |
| --- | --- | --- | --- | --- | --- | --- | --- | --- | --- | --- | --- | --- |
| 140 | 1 | 8.65 | 2.14 | 178 | 606 | 0 | 25 | 1 | 37 | 0 | 1 | 23.7 |
| 1 | 23 | 1.6 | 0.69 | 120 | 822 | 0 | 17 | 1 | 38 | 0 | 1 | 24.7 |
|  | 35 | 8.65 | 1.83 | 47.8 | 390 | 0 | 22 | 3 | 38 | 0 | 1 | 24.5 |
| 39 | 45 | 9.4 | 3.06 | 120 | 210 | 0 | 35 | 1 | 36 | 0 | 1 | 22.77 |
| 49 | 50 | 4.95 | 1.01 | 122 | 384 | 0 | 30 | 3 | 38 | 0 | 1 | 29.97 |
| 22 | 51 | 8.05 | 1.05 | 182 | 444 | 0 | 35 | 1 | 36 | 0 | 1 | 23.8 |
| 27 | 52 | 15.1 | 0.82 | 160 | 204 | 0 | 36 | 3 | 37 | 0 | 1 | 20.31 |
| 23 | 53 | 9.25 | 1.04 | 25.6 | 630 | 0 | 35 | 7 | 36 | 0 | 1 | 30.67 |
| 11 | 54 | 8.2 | 2.03 | 174 | 414 | 0 | 22 | 1 | 37 | 0 | 1 | 32.18 |
| 12 | 55 | 10.75 | 0.79 | 100 | 240 | 0 | 16 | 0 | 35 | 0 | 1 | 27.44 |
| 13 | 56 | 14.1 | 0.95 | 188 | 348 | 0 | 37 | 6 | 37 | 0 | 1 | 19.83 |
| 15 | 57 | 20 | 1.73 | 150 | 300 | 0 | 22 | 1 | 38 | 0 | 1 | 25.32 |
| 14 | 58 | 17.3 | 1.05 | 98 | 546 | 0 | 32 | 1 | 38 | 0 | 1 | 24.39 |
| 95 | 59 | 11.75 | 1.4 | 184 | 390 | 0 | 27 | 1 | 36 | 0 | 1 | 33.15 |
| 89 | 60 | 11.1 | 1.18 | 130 | 312 | 0 | 30 | 1 | 37 | 0 | 1 | 29.9 |
| 88 | 61 | 15.3 | 1.44 | 184 | 438 | 0 | 35 | 2 | 36 | 0 | 1 | 23.11 |
| 87 | 62 | 9.6 | 4.4 | 80 | 396 | 0 | 30 | 4 | 36 | 0 | 1 | 29.7 |
| 86 | 63 | 15.65 | 1.06 | 42 | 384 | 0 | 38 | 9 | 37 | 0 | 1 | 21.34 |
| 85 | 64 | 15.5 | 0.57 | 136 | 588 | 0 | 21 | 0 | 38 | 0 | 1 | 23.8 |
| 84 | 65 | 8.5 | 0.58 | 154 | 672 | 0 | 35 | 7 | 35 | 0 | 1 | 23.7 |
| 93 | 67 | 9.75 | 1.37 | 144 | 450 | 0 | 22 | 0 | 34 | 0 | 1 | 34.24 |
| 120 | 68 | 8 | 1.89 | 80 | 768 | 0 | 36 | 2 | 38 | 0 | 1 | 21.21 |
| 118 | 69 | 9.75 | 2.27 | 106 | 570 | 0 | 39 | 7 | 37 | 0 | 1 | 23.88 |
| 117 | 70 | 10.65 | 0.6 | 70 | 858 | 0 | 24 | 1 | 36 | 0 | 1 | 21.87 |
| 116 | 71 | 4.25 | 0.62 | 106 | 804 | 0 | 30 | 4 | 36 | 0 | 1 | 27.49 |
| 115 | 72 | 4.45 | 1.61 | 84 | 480 | 0 | 23 | 1 | 38 | 0 | 1 | 36.33 |
| 113 | 73 | 4.95 | 1.56 | 114 | 1266 | 0 | 23 | 0 | 36 | 0 | 1 | 23.8 |
| 112 | 74 | 6.05 | 1.36 | 82 | 486 | 0 | 22 | 0 | 38 | 0 | 1 | 17.21 |
| 111 | 75 | 5.55 | 1.3 | 132 | 546 | 0 | 24 | 0 | 38 | 0 | 1 | 22.3 |
| 104 | 77 | 6.45 | 1.61 | 164 | 516 | 0 | 19 | 0 | 38 | 0 | 1 | 23.53 |
| 106 | 78 | 5.9 | 1.53 | 78 | 372 | 0 | 30 | 2 | 36 | 0 | 1 | 29.37 |
| 103 | 80 | 11.3 | 2.29 | 138 | 522 | 0 | 33 | 4 | 37 | 0 | 1 | 27.18 |
| 102 | 81 | 5.05 | 0.98 | 108 | 486 | 0 | 23 | 0 | 36 | 0 | 1 | 24.22 |
| 101 | 82 | 8.35 | 1.17 | 96 | 438 | 0 | 25 | 0 | 36 | 0 | 1 | 24.73 |
| 100 | 83 | 6.45 | 0.76 | 66 | 366 | 0 | 23 | 2 | 36 | 0 | 1 | 34.96 |
| 155 | 84 | 5.15 | 1.14 | 140 | 204 | 0 | 30 | 4 | 37 | 0 | 1 | 26.3 |
| 151 | 85 | 6.5 | 0.99 | 140 | 240 | 0 | 32 | 4 | 37 | 0 | 1 | 28.03 |
| 150 | 86 | 6.2 | 1.29 | 92 | 426 | 0 | 20 | 0 | 37 | 0 | 1 | 24.8 |
| 149 | 87 | 11.05 | 0.74 | 70 | 336 | 0 | 29 | 7 | 38 | 0 | 1 | 31.91 |
| 148 | 88 | 8 | 0.93 | 134 | 228 | 0 | 35 | 5 | 36 | 0 | 1 | 22.77 |
| 147 | 89 | 6.25 | 1.67 | 82 | 306 | 0 | 38 | 11 | 38 | 0 | 1 | 34.58 |
| 144 | 90 | 6.7 | 0.89 | 186 | 264 | 0 | 29 | 4 | 36 | 0 | 1 | 23.23 |
| 142 | 91 | 7.2 | 1.37 | 88 | 378 | 0 | 28 | 2 | 38 | 0 | 1 | 29.37 |
| 141 | 92 | 6.5 | 1.19 | 72 | 456 | 0 | 20 | 0 | 37 | 0 | 1 | 27.18 |
| 139 | 93 | 7.15 | 1.24 | 80 | 408 | 0 | 26 | 4 | 36 | 0 | 1 | 24.22 |
| 132 | 96 | 11.25 | 1.11 | 78 | 486 | 0 | 20 | 0 | 39 | 0 | 1 | 33.33 |
| 131 | 97 | 11.25 | 0.88 | 116 | 396 | 0 | 37 | 9 | 37 | 0 | 1 | 26.67 |
| 130 | 98 | 11 | 0.34 | 118 | 462 | 0 | 39 | 6 | 37 | 0 | 1 | 35 |
| 129 | 99 | 14.7 | 0.84 | 112 | 426 | 0 | 30 | 2 | 38 | 0 | 1 | 31.59 |
| 128 | 100 | 13.3 | 0.58 | 120 | 474 | 0 | 39 | 5 | 36 | 0 | 1 | 21.45 |
| 135 | 6 | 8.4 | 1.21 | 154 | 714 | 1 | 30 | 5 | 37 | 1 | 0 | 36.51 |
| 134 | 7 | 6.8 | 2.18 | 202 | 570 | 1 | 20 | 2 | 37 | 1 | 0 |  |
| 107 | 15 | 5.7 | 1.96 | 126 | 468 | 1 | 23 | 0 | 38 | 1 | 0 | 25.71 |
| 99 | 16 | 10.45 | 2.06 | 124 | 438 | 1 | 28 | 1 | 40 | 1 | 0 | 26.5 |
| 3 | 21 | 2.3 | 0.9 | 110 | 486 | 1 | 28 | 5 | 36 | 1 | 0 | 24.22 |
| 92 | 24 | 2.4 | 1.75 | 156 | 564 | 1 | 23 | 1 | 36 | 1 | 0 | 33.33 |
| 90 | 26 | 1.8 | 1.61 | 90 | 744 | 1 | 28 | 3 | 40 | 1 | 0 | 26.67 |
| 6 | 38 | 9.9 | 1.94 | 56 | 288 | 1 | 26 | 6 | 37 | 1 | 0 | 35 |
| 145 | 2 | 8.7 | 2.07 | 118 | 558 | 2 | 26 | 0 | 38 | 1 | 0 | 31.59 |
| 138 | 3 | 9.5 | 1.18 | 158 | 444 | 2 | 30 | 1 | 37 | 1 | 0 | 24.8 |
| 137 | 4 | 10.15 | 1.6 | 166 | 564 | 2 | 19 | 0 | 37 | 1 | 0 | 26.3 |
| 136 | 5 | 8.3 | 1.53 | 162 | 444 | 2 | 27 | 0 | 37 | 1 | 0 | 28.63 |
| 133 | 8 | 9.9 | 2.06 | 104 | 486 | 2 | 30 | 4 | 36 | 1 | 0 | 26.8 |
| 127 | 9 | 10.4 | 0.96 | 250 | 510 | 2 | 35 | 4 | 37 | 1 | 0 | 28.13 |
| 126 | 10 | 9.4 | 1.37 | 190 | 450 | 2 | 28 | 0 | 36 | 1 | 0 | 38.29 |
| 121 | 11 | 6.35 | 2.56 | 152 | 912 | 2 |  | 3 | 37 | 1 | 0 | 25.6 |
| 119 | 12 | 7.35 | 2.34 | 193.8 | 450 | 2 | 28 | 0 | 36 | 1 | 0 | 22.49 |
| 114 | 13 | 7.95 | 1.51 | 146 | 504 | 2 | 19 | 0 | 38 | 1 | 0 | 21.93 |
| 108 | 14 | 8.65 | 1.57 | 102 | 402 | 2 | 20 | 0 | 37 | 1 | 0 | 24.22 |
| 98 | 17 | 6.9 | 0.92 | 124 | 354 | 2 | 28 | 1 | 37 | 1 | 0 | 33.33 |
| 97 | 18 | 10.3 | 1.13 | 90 | 462 | 2 | 35 | 9 | 36 | 1 | 0 | 26.67 |
| 96 | 19 | 2.7 | 1.78 | 68 | 474 | 2 | 20 | 1 | 36 | 1 | 0 | 35 |
| 152 | 20 | 6.05 | 3.1 | 58 | 414 | 2 | 20 | 0 | 37 | 1 | 0 | 31.59 |
| 2 | 22 | 2.65 | 0.81 | 170 | 948 | 2 | 36 | 1 | 36 | 1 | 0 | 26.67 |
| 91 | 25 | 2.85 | 1.7 | 134 | 408 | 2 | 37 | 7 | 38 | 1 | 0 | 35 |
| 125 | 27 | 3.05 | 1.68 | 84 | 384 | 2 | 30 | 3 | 36 | 1 | 0 | 31.59 |
| 124 | 28 | 3.8 | 0.64 | 74 | 546 | 2 | 20 | 3 | 36 | 1 | 0 | 24.22 |
| 123 | 29 | 9.9 | 0.82 | 164 | 756 | 2 | 19 | 0 | 36 | 1 | 0 | 33.33 |
| 122 | 30 | 6.65 | 1.74 | 56 | 732 | 2 | 38 | 4 | 38 | 1 | 0 | 26.67 |
| 36 | 31 | 7 | 0.92 | 124 | 432 | 2 | 21 | 0 | 37 | 1 | 0 | 35 |
| 30 | 32 | 9.35 | 0.86 | 90 | 2070 | 2 | 35 | 4 | 38 | 1 | 0 | 31.59 |
| 26 | 33 | 3.15 | 0.7 | 62 | 468 | 2 | 28 | 1 | 35 | 1 | 0 | 37.02 |
| 35 | 34 | 5.95 | 1.36 | 40 | 486 | 2 | 29 | 3 | 38 | 1 | 0 | 24.22 |
| 24 | 36 | 6.25 | 1.1 | 124 | 276 | 2 | 30 | 1 | 40 | 1 | 0 | 33.33 |
| 25 | 37 | 5.85 | 3.34 | 44 | 294 | 2 | 23 | 1 | 38 | 1 | 0 | 26.67 |
| 5 | 39 | 7.6 | 1.26 | 122 | 414 | 2 | 38 | 0 | 37 | 1 | 0 | 35 |
| 4 | 40 | 8.3 | 1.32 | 98 | 582 | 2 | 35 | 7 | 36 | 1 | 0 | 31.59 |
| 77 | 41 | 7.65 | 1.23 | 102 | 312 | 2 | 28 | 9 | 38 | 1 | 0 | 24.8 |
| 63 | 42 | 7.35 | 3.21 | 54 | 3090 | 2 | 36 | 0 | 37 | 1 | 0 | 23.4 |
| 62 | 43 | 11.25 | 2.18 | 102 | 444 | 2 | 31 | 4 | 37 | 1 | 0 | 25.39 |
| 37 | 44 | 9.7 | 2.48 | 182 | 360 | 2 | 18 | 0 | 36 | 1 | 0 | 26.3 |
| 47 | 46 | 8.2 | 1.22 | 148 | 396 | 2 | 35 | 3 | 37 | 1 | 0 | 27.55 |
| 46 | 47 | 8.5 | 1.41 | 184 | 378 | 2 | 32 | 2 | 38 | 1 | 0 | 36.68 |
| 45 | 48 | 9.2 | 1.99 | 126 | 114 | 2 | 37 | 1 | 38 | 1 | 0 | 24.22 |
| 44 | 49 | 10.5 | 2.34 | 148 | 192 | 2 | 38 | 4 | 38 | 1 | 0 | 36.2 |
|  | 66 | 11.95 | 0.92 | 108 | 588 | 2 | 18 | 3 | 37 | 1 | 0 | 24.22 |
|  | 76 | 6.05 | 1.45 | 124 | 498 | 2 | 35 | 2 | 38 | 1 | 0 | 33.33 |
|  | 79 | 6.1 | 2.06 | 134 | 528 | 2 | 32 | 1 | 38 | 1 | 0 | 26.67 |
|  | 94 | 8.5 | 1.24 | 60 | 354 | 2 | 37 | 4 | 36 | 1 | 0 | 35 |
|  | 95 | 7.2 | 3.56 | 82 | 372 | 2 | 38 | 2 | 37 | 1 | 0 | 31.59 |
